# The Prognostic Significance of Pontine-White Matter Score in Primary Central Nervous System Lymphoma Patients

**DOI:** 10.3390/cancers16152708

**Published:** 2024-07-30

**Authors:** Yongjiang Li, Yiwen Mo, Mingshi Chen, Wenbiao Zhang, Shuangjiang Li, Xu Zhang

**Affiliations:** 1 Department of Nuclear Medicine, Sichuan Clinical Research Center for Cancer, Sichuan Cancer Hospital and Institute, Sichuan Cancer Center, Affiliated Cancer Hospital of University of Electronic Science and Technology of China, Chengdu 610041, China; 2 Department of Nuclear Medicine, Sun Yat-sen University Cancer Center, Guangzhou 510060, China; 3 Department of Radiology and Nuclear Medicine, Amsterdam University Medical Center, University of Amsterdam, 1105 AZ Amsterdam, The Netherlands; 4 Department of Biomedical Engineering and Physics, Amsterdam University Medical Center, 1105 AZ Amsterdam, The Netherlands; 5 Department of Radiology, Sun Yat-sen University Cancer Center, Guangzhou 510060, China; 6 Department of Endoscopy, Sun Yat-sen University Cancer Center, Guangzhou 510060, China

**Keywords:** CNS lymphoma, ^18^F-FDG PET, prognosis, therapeutic evaluation

## Abstract

**Simple Summary:**

This study explored the prognostic significance of the pontine-white matter (PW) score in primary central nervous system (CNS) lymphoma patients with post-treatment ^18^F-FDG PET/CT and PET/MR imaging. Eligible patients were enrolled from January 2014 to December 2022. The PW score, derived from FDG uptake of the pons and white matter, was used to evaluate the metabolic activity of the treated lesion and its prognostic implications. A total of 90 patients across PET/CT and PET/MR modalities were assessed. The PW score demonstrated a robust discriminative ability in identifying patients with worse outcomes. It was also found to be a significant and independent indicator for worse prognosis in both PET/CT and PET/MR groups. The study demonstrated that this novel internal standardization indicator was an effective tool for risk stratification in primary CNS lymphoma post-treatment scenarios.

**Abstract:**

Background: Limited data exist on the significance of PET imaging and quantitative PET parameters in primary central nervous system (CNS) lymphoma due to its relative rarity. This study was conducted to investigate the prognostic value of a novel internal standardization indicator, the pontine-white matter (PW) score, in primary CNS lymphoma patients undergoing post-treatment ^18^F-FDG PET/CT and PET/MR imaging. Methods: From January 2014 to December 2022, eligible patients with primary CNS lymphoma who underwent post-treatment PET imaging were enrolled. Using the FDG uptake of the pons and white matter as an internal reference, the PW score was graded based on the metabolism of the post-therapeutic lesion for each patient, and its associations with patients’ prognosis were investigated. Results: In total, 41 patients with post-treatment PET/CT and 49 patients with post-treatment PET/MR imaging were enrolled. ROC curve analysis indicated that the PW score possessed robust discriminative ability in distinguishing patients with worse outcomes. Furthermore, a higher PW score was significantly correlated with and identified as an independent prognostic indicator for, worse prognosis in both the PET/CT and PET/MR cohorts. Conclusion: The study demonstrated that the PW score was an effective prognostic indicator for identifying post-treatment primary CNS lymphoma patients with worse outcomes.

## 1. Introduction

Primary central nervous system (CNS) lymphoma is a rare but aggressive type of extra-nodal non-Hodgkin lymphoma that is confined to the CNS compartment at diagnosis. In immunocompetent patients, it accounts for about 4% of all intracranial neoplasms and 4–6% of all extra-nodal lymphomas [1,2]. Histologically, primary diffuse large B-cell lymphoma (DLBCL) of the CNS is recognized as a distinct entity in the WHO classification of lymphoid neoplasms, accounting for 90% of all primary CNS lymphomas. Occasionally, primary CNS lymphoma may also present as Burkitt lymphoma, low-grade lymphoma, or T-cell lymphoma [3,4,5]. The standard diagnostic procedure for primary CNS lymphoma involves histopathological confirmation through a stereotactic biopsy of the intracranial lesion [6].

Unlike other intracranial neoplasms, primary CNS lymphoma responds favorably to high-dose methotrexate (HD-MTX)-based chemotherapy, which is also the standard treatment recommended by clinical practice guidelines [7,8,9]. However, survival rates are still lower compared to lymphomas not involving the CNS, with only half of the patients achieving durable remissions. The prognosis of the non-responders to the first-line chemotherapy remains poor [10,11].

Although limited data have been established on the value of positron emission tomography (PET) imaging and quantitative PET parameters for CNS lymphoma, a consensus has been reached that ^18^F-fluorodeoxyglucose (FDG) PET/CT could serve as a practical option for the diagnosis, post-therapeutic response evaluation, and relapse monitoring of CNS lymphoma during follow-up. This utility is due to the extremely high and homogeneous FDG uptake of this type of lesion compared to other intracranial tumors, such as high-grade gliomas and metastases [12,13]. Although the cortical brain exhibits high FDG uptake, most primary CNS lymphomas are actually located in the white matter, which potentially reduces interference in the detection of these FDG-avid intracranial lesions [14,15,16]. Kawai et al. identified that high maximum standard uptake values (SUVmax) were correlated with worse progression-free survival and overall survival in univariate analyses [17]. Additionally, Yamaguchi et al. suggested that the ratio of tumor activity to normal contralateral cortex activity is a superior indicator to SUVmax for detecting primary CNS lymphoma [18]. However, the number of patients with primary CNS lymphoma in these studies was relatively small (*n* = 17 and *n* = 19, respectively), and the potentially more suitable PET imaging facility for primary CNS lymphoma [19], the PET/MR, has not yet been investigated.

The present study investigated the potential prognostic value of post-treatment ^18^F-FDG PET/CT and PET/MR in patients with primary CNS lymphoma. Using normal intracranial structures, the pons and white matter, as internal references, we proposed a simple visual metabolic score, the Pontine-white matter score (PW Score), as an indicator of lymphoma treatment response.

## 2. Materials and Methods

### 2.1. Cohort Selection

The study was examined and approved by the Institutional Review Board and the Medical Ethics Committee of Sun Yat-sen Cancer Center. Patients with primary CNS lymphoma admitted to our institution between January 2014 and December 2022 with end-of-treatment PET/CT or PET/MR examination were enrolled in the study. The inclusion criteria were: (1) biopsy-verified primary CNS lymphoma; (2) exclusion of systemic lymphoma and concomitant malignancy; (3) absence of leptomeningeal lesion or eye involvement; (4) exclusion of HIV infection; (5) age ≥ 15 years; (6) PET/CT or PET/MR conducted within 8 weeks after the last dose of chemotherapy. The patients with incomplete treatment and follow-up data at our institution were excluded.

### 2.2. PET/CT and PET/MR Imaging

All patients fasted for 6 h before ^18^F-FDG administration, and their blood glucose level was checked to be stable and below 200 mg/dL (11.1 mmol/L). PET/CT scans were performed with integrated PET/CT scanners (Biograph mCT, Siemens Healthcare, Henkestr, Germany, or uEXPLORER, United Imaging Healthcare, Shanghai, China), and PET/MR scans were conducted with uPMR 790 scanner (United Imaging Healthcare, Shanghai, China). Scans were conducted 60 min after ^18^F-FDG injection (0.1 mCi/kg or 3.7 MBq/kg body weight).

For the PET/CT imaging, CT scans of the whole body from skull to mid-thigh were obtained without contrast enhancement for attenuation correction and fusion (80–200 mAs, 120 kVp, 3 mm slice thickness for the Biograph mCT scanner, and 2.89 mm slice thickness for the uEXPLORER scanner), and were reconstructed in a 512 × 512 matrix. The subsequent PET scan was conducted and reconstructed with a slice thickness of 2 mm, using the Ordered Subsets Expectation Maximization (OSEM) iterative reconstruction method.

For the PET/MR scan, the images were reconstructed using the OSEM algorithm, which incorporated 20 subsets and 2 iterations, along with point spread function (PSF) and time-of-flight (TOF) modeling. The resulting matrix was 256 × 256 × 113, with each voxel measuring 2.4 × 2.4 × 2.85 mm^3^. The MR imaging protocols encompassed T1-weighted spin-echo sequence, T2-weighted imaging, fluid-attenuated inversion recovery (FLAIR) imaging, diffusion-weighted imaging (DWI), and apparent diffusion coefficient (ADC) mapping.

### 2.3. Image Analysis

The image analysis of the PET/CT and PET/MR images was conducted by two experienced nuclear medicine physicians through visual and semiquantitative evaluation, focusing on predefined regions of interest (ROIs). The ROIs were selected to include areas of lesions as well as reference regions including the white matter and pons for comparative purposes. The PW score was graded according to the following principle: if the lesion’s uptake was greater than that of the pons, it was scored as 2 points; if the lesion’s uptake was between that of the pons and the white matter, it was scored as 1 point; and if the lesion’s uptake was less than that of the white matter, it was scored as 0 points.

### 2.4. Statistical Analysis

Statistical analyses were conducted using SPSS Statistics version 22.0 (IBM Corp., Chicago, IL, USA). Receiver operating characteristic (ROC) analyses were conducted using 2-year disease progression and overall survival as end-points, and the corresponding areas under the curves (AUCs) were calculated. The survival curves were generated using the Kaplan–Meier analysis and evaluated with the log-rank test. Indicators with *p* < 0.1 in the univariate analysis were included in the multivariate analysis. Univariate and multivariate analyses for progression-free survival (PFS) and overall survival (OS) were performed using the Cox regression model to identify independent prognostic indicators.

## 3. Results

### 3.1. Patient Characteristics

A total of 41 patients were enrolled in the PET/CT cohort and 49 patients were enrolled in the PET/MR cohort. The characteristics of the patients are summarized in Table 1. The PET/CT cohort had 21 male patients (51.2%), and 19 patients (46.3%) with age over 60. The PET/MR had 26 male patients (53.1%), and 29 patients (59.2%) with age over 60. Multiple lesions were presented in 22 patients (53.7%) of the PET/CT cohort, and 31 patients (63.3%) of the PET/MR cohort. The ECGO score was 0–1 point for 20 patients (48.8%) in the PET/CT cohort and 27 patients (55.1%) in the PET/MR cohort. All the patients received high-dose methotrexate based chemotherapy, whole-brain radiotherapy was given in 19 patients (46.3%) of the PET/CT cohort and 7 patients (14.3%) of the PET/MR cohort, and autologous stem cell transplantation (ASCT) was given in 7 patients (17.1%) of the PET/CT cohort and 10 patients (20.4%) of the PET/MR cohort. In total, 28 patients (68.3%), 8 patients (19.5%) and 5 patients (12.2%) had the PW score of 0, 1 and 2 points, respectively; while 30 patients (61.2%), 15 patients (30.6%) and 4 patients (8.2%) had the PW score of 0, 1 and 2 points, respectively.

### 3.2. ROC Curve Analysis

**Figure 1:**
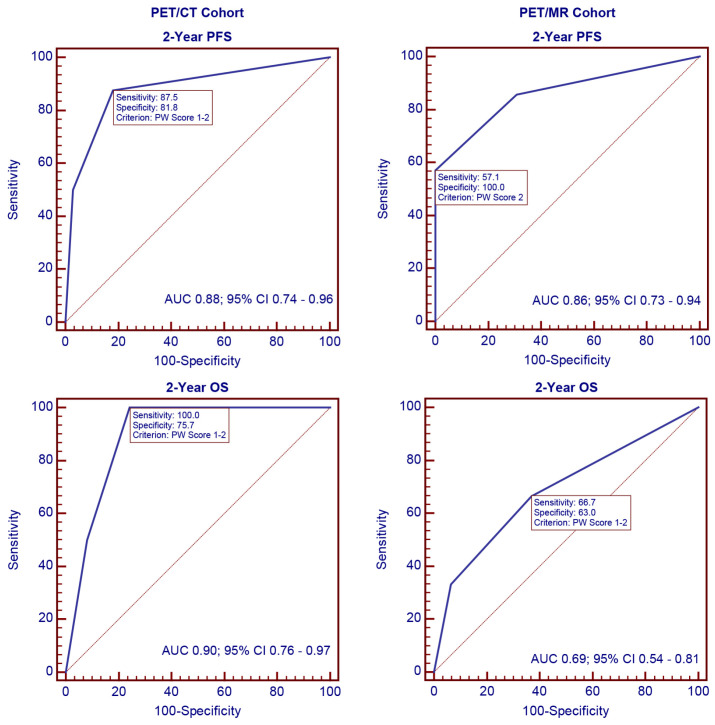
ROC analysis of the PW score for 2-year disease progression and overall survival.

ROC curves were generated to evaluate the discriminatory ability of the PW score for 2-year disease progression and overall survival. As shown in Figure 1, the AUC values for 2-year disease progression were 0.88 (95% confidence interval (CI) 0.74–0.96; *p* < 0.001) in the PET/CT cohort and 0.86 (95% CI 0.73–0.94; *p* = 0.001) in the PET/MR cohort. For 2-year overall survival, the AUC values were 0.90 (95% CI 0.76–0.97; *p* < 0.001) in the PET/CT cohort and 0.69 (95% CI 0.54–0.81; *p* = 0.338) in the PET/MR cohort.

### 3.3. Survival Curve Analysis for PFS in PET/CT and PET/MR Cohorts

**Figure 2:**
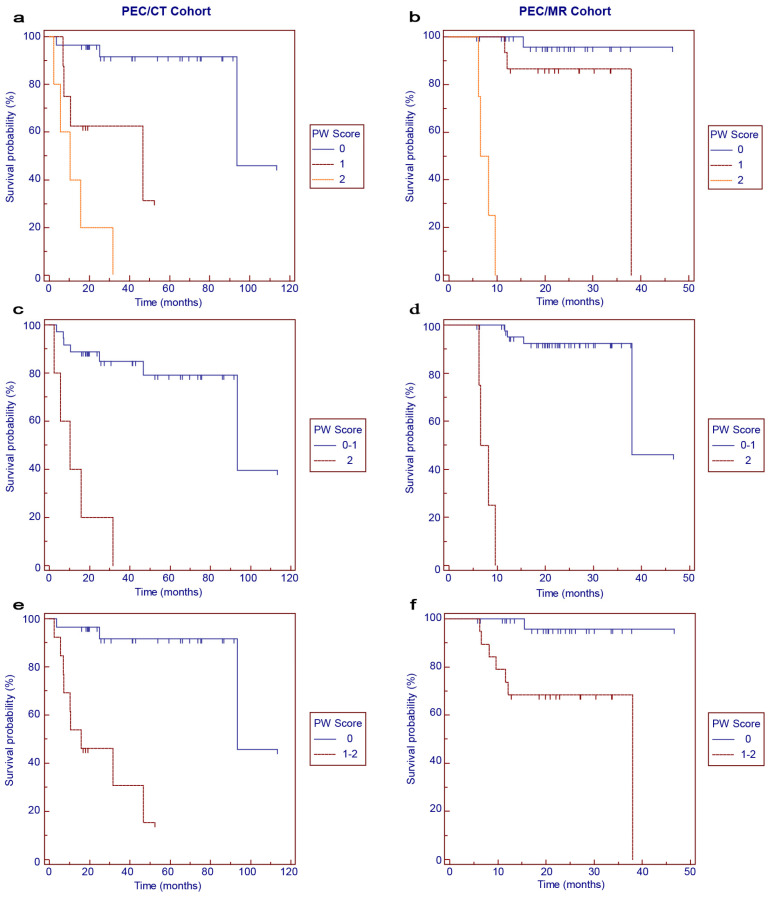
Kaplan–Meier survival curves for progression-free survival in the PET/CT (**a**,**c**,**e**) and PET/MR (**b**,**d**,**f**) cohorts according to PW scores.

During the follow-up period, disease progression occurred in 12 patients (29.3%) and 8 patients (16.3%) of the PET/CT and PET/MR cohorts, respectively. The survival curves for PFS according to the PW scores are shown in Figure 2. In general, worse PFS was correlated with higher PW scores (Figure 2a,b, *p* < 0.001). When the 3-point scale was categorized into binary subgroups, PW score 2 was significantly associated with worse PFS in the PET/CT cohort (Figure 2c, *p* < 0.001) and PET/MR cohort (Figure 2d, *p* < 0.001) compared with PW score 0–1. Similarly, patients with PW score 1–2 had significantly worse PFS in the PET/CT cohort (Figure 2e, *p* < 0.001) and PET/MR cohort (Figure 2f, *p* = 0.004) than those with PW score 0.

### 3.4. Survival Curve Analysis for OS in PET/CT and PET/MR Cohorts

**Figure 3:**
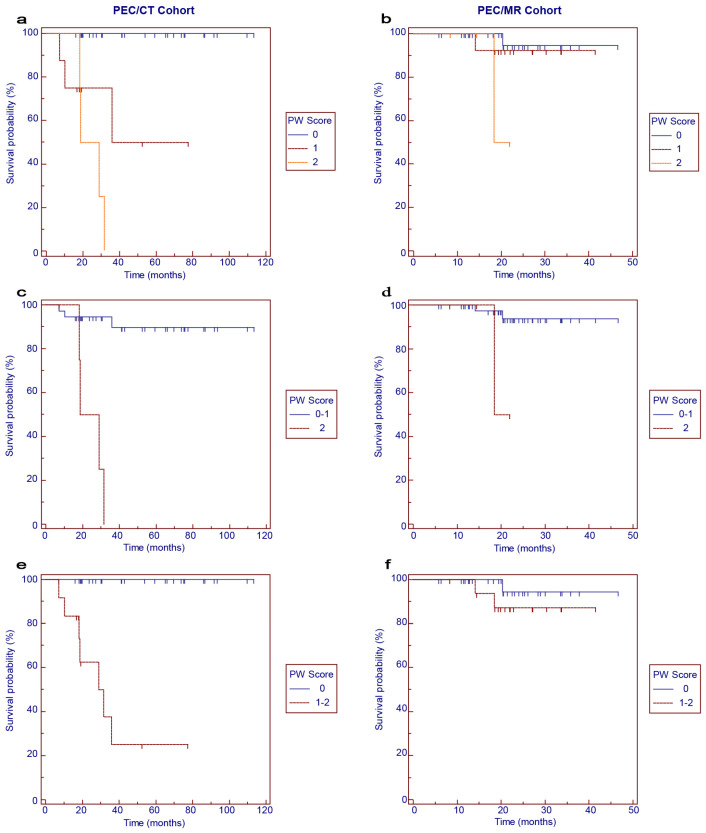
Kaplan–Meier survival curves for overall survival in the PET/CT (**a**,**c**,**e**) and PET/MR (**b**,**d**,**f**) cohorts according to PW scores.

The survival curves for OS according to the PW scores are shown in Figure 3. In the PET/CT cohort, worse OS was correlated with higher PW scores (Figure 3a, *p* < 0.001). When the 3-point scale was categorized into binary subgroups, PW score 2 was significantly associated with worse OS than PW score 0–1 (Figure 3c, *p* < 0.001), and PW score 1–2 was significantly associated with worse OS than PW score 0 (Figure 3e, *p* < 0.001). In the PET/MR cohort, a trend of worse OS associated with higher PW scores could be observed, but the significance was not reached (Figure 3b,d,f).

### 3.5. Univariate and Multivariate Analysis for PFS and OS in PET/CT and PET/MR Cohorts

The univariate analysis for PFS in the PET/CT and PET/MR cohorts was presented in Table 2. The PW score was significantly correlated with worse PFS in the PET/CT cohort (hazard ratio (HR) 5.34, *p* < 0.001), while radiotherapy (HR 5.53, *p* = 0.026) and the PW score (HR 32.80, *p* = 0.001) was significantly associated with worse PFS in the PET/MR cohort.

The univariate analysis for OS in the PET/CT and PET/MR cohorts was presented in Table 3. The PW score was significantly correlated with worse OS in the PET/CT cohort (HR 10.20, *p* = 0.001), and no indicator was found to be significantly associated with worse OS in the PET/MR cohort.

The results of the multivariate analysis are shown in Table 4. For PFS, the PW score was identified as the independent prognostic indicator in the PET/CT cohort (HR 5.34, *p* < 0.001) and PET/MR cohort (HR 25.66, *p* = 0.009). For OS, the PW score was identified as the independent prognostic indicator in the PET/CT cohort (HR 9.01, *p* = 0.001), and no significant indicator was identified in the PET/MR cohort.

## 4. Discussion

In this study, we first introduced the PW score as a novel and effective prognostic indicator in patients with primary CNS lymphoma receiving post-treatment PET/CT imaging. Additionally, we validated its effectiveness in predicting disease progression within the PET/MR cohort.

Due to the enhanced soft tissue resolution and multiple imaging sequences of MR, the International Primary CNS Lymphoma Collaborative Group (IPCG) has recommended MR as an essential imaging modality for the treatment of primary CNS lymphoma [20]. Although ^18^F-FDG PET/MR use in primary CNS lymphoma has yet to be systematically investigated, it is reasonable to infer that PET/MR imaging, which integrates the strengths of both PET and MR, will significantly impact the management of these patients. The imaging analysis focused on white matter, providing detailed insights into uptake patterns relevant to primary CNS lymphoma. Since MR scans generally take longer to complete than CT scans, in clinical practice, PET/MR is often used for localized scans rather than the whole-body scans typically performed in PET/CT. The PW score, instead of referencing extracranial structures requiring whole-body PET imaging, relies on FDG uptake in the pons and white matter. These regions can be captured simultaneously in a regional PET scan of the head, making this method particularly suitable for PET/MR imaging that targets specific areas.

The utility of the PW score necessitates a consistent physiological metabolism in normal intracranial structures to ensure comparability among individuals. Sprinz et al. demonstrated that higher blood glucose levels significantly reduce FDG uptake in the brain [21], and that the time interval can also impact FDG uptake in normal organs [22]. Moreover, cerebral glucose metabolism in normal individuals can be affected by recent administration of chemotherapy, caffeine, alcohol, amphetamines, cocaine, anesthetics, benzodiazepines, and other psychotropic drugs [23,24,25]. In our cohorts, blood glucose levels were strictly monitored before each scan, excluding patients with unsuitable glucose levels from PET imaging. Additionally, other factors such as the time interval and resting period before the PET scan were controlled according to clinical routine, and recent use of specific drugs was checked, ensuring that the FDG uptake of the referenced structures was minimally influenced by these physiological and pharmacological factors.

Although SUVs and metabolic tumor burden measured on ^18^F-FDG PET/CT have been recognized as prognostic indicators in patients with systemic lymphoma, data on primary CNS lymphoma remain limited and primarily explored retrospectively in a few studies. Albano et al. found an inverse correlation between higher metabolic tumor burden on pre-treatment ^18^F-FDG PET/CT and prognosis in a cohort of 52 primary CNS lymphoma patients [15]. In another study involving 53 primary CNS lymphoma patients receiving HD-MTX and ibrutinib combination therapy, Kerbs et al. observed that higher metabolic parameters significantly correlated with worse prognosis, with the sum of SUVmax emerging as a robust independent indicator [13]. On the other hand, Kasenda et al. suggested that measurement through internal standardization (using a reference region) would be a more robust approach and better suited for inter-individual comparison in primary CNS lymphoma patients than using SUVs [26]. This is because SUV calculations can be cumbersome and potentially error-prone due to factors such as variations in injected dose calculations, body weight assessments, and definitions of ROIs.

The ratio of tumor to normal contralateral cortex (T/N ratio) has been implicated as a method of internal standardization in the diagnosis of CNS lymphoma compared to other malignant brain tumors, which is more reliable than SUVmax for differential diagnosis [18]. Additionally, this ratio is minimally influenced by individual factors such as plasma glucose level, age, body weight, and dosage level. Similarly, another semiquantitative visual rating scale, which uses the physiological FDG uptake of the cerebellum as the baseline reference region and rates the metabolism of CNS lymphoma lesions on a 10-point scale linearly related to SUVmax, showed a significant inverse correlation between a rating over 3 points and patient prognosis [26]. In our study, the PW score, based on the FDG uptake of the pontine and white matter, was significantly associated with a worse prognosis in both PET/CT and PET/MR cohorts. Furthermore, whether the 3-point PW score was categorized by a cutoff of PW0–1/2 or PW0/1–2, it demonstrated favorable discriminative ability among groups of primary CNS lymphoma patients, indicating its potential usability in clinical applications.

Our study had some limitations. Firstly, it included only primary CNS patients with the histological type of aggressive B-cell lymphoma, which constitutes the majority of all primary CNS lymphomas. The applicability of the PW score to other histological types of CNS lymphoma remains to be further investigated. Secondly, the use of PET/CT or PET/MR scans may lead to the preferential inclusion of patients who are more compliant and financially stable. Thirdly, although this is currently the largest study investigating the role of ^18^F-FDG PET imaging in primary CNS lymphoma, the sample size remains small due to the rarity of the disease. The results should be interpreted with caution and require further validation.

## 5. Conclusions

In conclusion, the study demonstrated that the PW score was a novel and effective prognostic indicator for patients with primary CNS lymphoma receiving post-treatment PET/CT, which also exhibited satisfactory discriminatory ability in predicting disease progression in those undergoing post-treatment PET/MR.

## Figures and Tables

**Table 1:** Patient characteristics.

Variables		PET/CT Cohort (%)	PET/MR Cohort (%)
Total number		41 (100)	49 (100)
Gender	Male	21 (51.2)	26 (53.1)
	Female	20 (48.8)	23 (46.9)
Age	≥60	19 (46.3)	29 (59.2)
	<60	22 (53.7)	20 (40.8)
Number of tumor lesions	Multiple	22 (53.7)	31 (63.3)
	Single	19 (46.3)	18 (36.7)
Pathology	DLBLC	38 (92.7)	48 (98.0)
	HDBLC	3 (7.3)	1 (2.0)
Radiotherapy	Yes	19 (46.3)	7 (14.3)
	No	22 (53.7)	42 (85.7)
ASCT	Yes	7 (17.1)	10 (20.4)
	No	34 (82.9)	39 (79.6)
ECGO	0–1	20 (48.8)	27 (55.1)
	2–3	21 (51.2)	22 (44.9)
PW Score	0	28 (68.3)	30 (61.2)
	1	8 (19.5)	15 (30.6)
	2	5 (12.2)	4 (8.2)
Progression	Absence	29 (70.7)	41 (83.7)
	Presence	12 (29.3)	8 (16.3)
Survival	Alive	34 (82.9)	46 (93.9)
	Dead	7 (17.1)	3 (6.1)

Abbreviations: DLBCL, diffuse large B cell lymphoma; HDBCL, high-grade large B cell lymphoma; ASCT, autologous stem cell transplantation; ECOG, Eastern Cooperative Oncology Group.

**Table 2:** Univariate analysis for progression-free survival in PET/CT and PET/MR cohorts.

Variables	Univariate Analysis		
	HR	95% CI	*p*-Value
**PET/CT cohort (Progression-free survival)**			
Age (≥60 vs. <60)	1.06	0.32–3.47	0.930
Gender (Male vs. Female)	0.82	0.25–2.69	0.745
Lesion number (Multiple vs. Single)	2.91	0.79–10.76	0.110
ECGO (2–3 vs. 0–1)	1.45	0.46–4.60	0.530
Radiotherapy	0.77	0.23–2.55	0.673
ASCT	0.94	0.20–4.37	0.940
PW Score	5.34	2.48–11.49	<0.001 *
**PET/MR cohort (Progression-free survival)**			
Age (≥ 60 vs. <60)	0.48	0.11–2.15	0.338
Gender (Male vs. Female)	0.66	0.15–2.96	0.590
Lesion number (Multiple vs. Single)	1.94	0.39–9.68	0.417
ECGO (2–3 vs. 0–1)	1.25	0.31–5.01	0.753
Radiotherapy	5.53	1.23–24.82	0.026 *
ASCT	2.77	0.62–12.38	0.183
PW Score	32.80	4.06–265.34	0.001 *

* *p* < 0.05. Abbreviations: ECOG, Eastern Cooperative Oncology Group; ASCT, autologous stem cell transplantation.

**Table 3:** Univariate analysis for overall survival in PET/CT and PET/MR cohorts.

Variables	Univariate Analysis		
	HR	95% CI	*p*-Value
**PET/CT cohort (Overall survival)**			
Age (≥60 vs. <60)	0.48	0.09–2.49	0.382
Gender (Male vs. Female)	1.36	0.30–6.07	0.690
Lesion number (Multiple vs. Single)	6.48	0.78–53.98	0.084
ECGO (2–3 vs. 0–1)	0.86	0.19–3.87	0.845
Radiotherapy	1.12	0.25–5.08	0.875
ASCT	0.59	0.07–4.92	0.622
PW Score	10.20	2.74–37.90	0.001 *
**PET/MR cohort (Overall survival)**			
Age (≥60 vs. <60)	N/A		0.401
Gender (Male vs. Female)	0.56	0.05–6.21	0.639
Lesion number (Multiple vs. Single)	N/A		0.477
ECGO (2–3 vs. 0–1)	N/A		0.419
Radiotherapy	3.67	0.32–41.95	0.295
ASCT	6.17	0.56–68.17	0.137
PW Score	3.33	0.69–16.05	0.134

* *p* < 0.05; N/A, not available due to a wide range of 95% CI. Abbreviations: ECOG, Eastern Cooperative Oncology Group; ASCT, autologous stem cell transplantation.

**Table 4:** Multivariate analysis for progression-free survival and overall survival in PET/CT and PET/MR cohorts.

Variables	Multivariate Analysis		
	HR	95% CI	*p*-Value
**Progression-free survival**			
**PET/CT cohort**			
PW Score	5.34	2.48–11.49	<0.001 *
**PET/MR cohort**			
Radiotherapy	1.55	0.11–21.26	0.742
PW Score	25.66	2.21–297.78	0.009 *
**Overall survival**			
**PET/CT cohort**			
Lesion number	2.94	0.34–25.73	0.329
PW Score	9.01	2.37–34.23	0.001 *
**PET/MR cohort**			
No candidate indicators available.			

* *p* < 0.05.

## Data Availability

The datasets used and/or analyzed in this study are available from the corresponding author upon reasonable request.
